# Accuracy is not enough: explainable boosting machine model and identification of candidate biomarkers for real-time sepsis risk assessment in the emergency department

**DOI:** 10.1186/s12873-025-01402-w

**Published:** 2025-11-28

**Authors:** Fatma Hilal Yagin, Umran Aygun, Cemil Colak, Amal K. Alkhalifa, Sarah A. Alzakari, Mohammadreza Aghaei

**Affiliations:** 1 Department of Biostatistics, Faculty of Medicine, Malatya Turgut Ozal University, Malatya, 44280 Türkiye; 2 Department of Computer Science, Lakehead University, Thunder Bay, ON P7B 5E1 Canada; 3 Department of Anesthesiology and Reanimation, Malatya Yesilyurt Hasan Calık State Hospital, Malatya, 44929 Türkiye; 4 Department of Biostatistics and Medical Informatics, Faculty of Medicine, Inonu University, Malatya, 44280 Türkiye; 5 Department of Computer Sciences, College of Computer and Information Sciences, Princess Nourah Bint Abdulrahman University, P.O. Box 84428, Riyadh, 11671 Saudi Arabia; 6 Department of Ocean Operations and Civil Engineering, Norwegian University of Science and Technology (NTNU), Alesund, Norway; 7 Department of Sustainable Systems Engineering (INATECH), Albert Ludwigs University of Freiburg, Freiburg, Germany

**Keywords:** Sepsis, Machine learning, Explainable artificial intelligence, Explainable boosting machine, Biomarker

## Abstract

**Background:**

Sepsis poses a significant threat in emergency settings, necessitating tools for early and interpretable risk assessment. This study aimed to develop a robust explainable boosting machine (EBM) model, one of the explainable artificial intelligence (XAI) technologies, to construct a predictive model that balances high accuracy and clinical interpretability for use in emergency departments (EDs) and to examine candidate biomarkers.

**Methods:**

The study identified a significant class imbalance problem in the sepsis distribution among 560 sepsis and 1012 non-sepsis patients. To address the imbalance issue, SMOTE-NC was applied in the training data. The data was divided into two parts, 80% training and 20% testing. To ensure the reliability of the models and to report unbiased results, this process was repeated 100 times and the average performance was reported. To determine the best model for sepsis prediction, five different models (AdaBoost, Gradient Boosting, CatBoost, LightGBM, and EBM) were trained, and their performances were evaluated. In the last stage, we presented local and global explanations of EBM.

**Results:**

The EBM model achieved the highest success by reaching 79.1% F1-score, 80.9% sensitivity, and 84.8% AUC after resampling. In the global explanations, the variables with the highest weights in the model’s decision process were identified as positive blood culture, oxygen saturation, and procalcitonin, respectively.

**Conclusion:**

The EBM model accurately predicts sepsis risk based on clinically relevant biomarkers. The model’s high performance and inherent transparency can foster trust among clinicians and facilitate its integration into emergency department workflows for real-time decision support.

## Introduction

Sepsis, a life-threatening systemic infection response, remains a critical global health issue with high mortality rates, demanding early diagnosis for improved patient outcomes. This study aims to develop an explainable machine learning model for early sepsis risk prediction in emergency departments. Significant physiological and laboratory differences were observed between sepsis and control groups, including elevated inflammatory markers and vital signs. With approximately 45 million annual cases and 11 million deaths worldwide, sepsis accounts for nearly 20% of global mortality, highlighting the urgent need for advanced predictive tools to enhance clinical decision-making and reduce sepsis-related fatalities through timely intervention [1]. In current clinical practice, the diagnosis of sepsis is often based on criteria such as SIRS, SOFA, and qSOFA scores; however, the specificity and sensitivity of these approaches may be limited [2]. In addition, biomarkers—such as C-reactive protein (CRP), procalcitonin (PCT), and plasma lactate—play an important role in the diagnosis and prognosis of sepsis, but they may not be sensitive enough to be an early and reliable marker on their own [3, 4]. However, due to the varying clinical presentations and biomarker dynamics from patient to patient, multidimensional data analysis has become a necessity [3, 4]. Machine learning shows great promise in sepsis prediction through tree-based and ensemble methods. However, their “black box” nature limits clinical acceptance. Explainable Artificial Intelligence (XAI) addresses this by making model decisions transparent, thereby increasing clinician trust and facilitating the adoption of decision support systems [5, 6]. Explainable Boosting Machine (EBM) is an XAI method that offers both high accuracy and understandability. EBM combines security and transparency with shape functions that graphically reveal the contribution of each feature to the decision process [5]. A recent scoping review systematically demonstrated the contribution of feature engineering strategies to performance metrics such as AUROC, sensitivity, and specificity in sepsis prediction models [7]. In addition, Liu et al. [8] demonstrated that dataset diversity strengthens model generalizability by achieving AUC of 0.807 and 0.762 in internal and external validation with the explainable XGBoost model they developed in intensive care patients with traumatic brain injury. Similarly, the interpretable model developed by He and Qiu [9] on the MIMIC-III database provided high calibration and fairness criteria in 28-day mortality prediction with SHAP values. These studies highlight that not only algorithm selection but also integrating data engineering, explainability methods, and model fairness are critical to the success of sepsis early warning systems.

Despite the growing utilization of artificial intelligence for early sepsis detection, the clinical translation of machine learning models faces challenges including lack of standardization, variability in study designs, and limited external validation. Furthermore, many high-performing models are trained on intensive care unit (ICU) datasets (e.g., MIMIC-III), which may not generalize well to the dynamic, high-pressure environment of the emergency department (ED) where patient acuity, data availability, and the urgency of decision-making differ significantly. This gap is critical, as ED clinicians require decision support tools that are not only accurate but also interpretable, providing actionable and clinically relevant explanations to build trust and facilitate adoption [10, 11]. Zhou et al. [12] utilized SHAP values and provided insights into how different clinical features influenced the risk predictions. This level of interpretability aids clinicians in understanding the rationale behind risk assessments, thereby aligning model outputs with clinical reasoning. The integration of interpretable machine learning models tailored to emergency department populations is not merely a technical advancement but a clinical necessity. Therefore, this study aims to develop and validate an interpretable and robust EBM model specifically adapted to the emergency department setting, addressing the problem of class imbalance for early prediction of sepsis risk and examining clinical biomarker candidates affecting sepsis.

## Materials and methods

### Materials

This study was conducted using open access data from a prospective observational study in adults with and without sepsis. The study included all patients aged 18 years and older presenting to the emergency department with suspected sepsis. In the sample examined in the study, 560 of 1,572 adult patients were positive for sepsis and 1,012 were negative. We examined the diagnostic performance of sepsis biomarkers alone and in combination for the confirmed diagnosis of sepsis using sepsis-3 criteria. The study used outcome measures to assess the presence and absence of sepsis based on sepsis-3 criteria. According to Sepsis-3, Organ dysfunction, is defined as a sudden change of at least two points in the total SOFA score due to infection. In patients previously unknown to have organ dysfunction, the initial SOFA score was zero. Confirmed bacterial infection for sepsis-3 demonstrated clinical infection, identification of associated bacteria by culture, and a positive blood culture for bacteremia. The Inonu University Non-Interventional Clinical Research Institutional Review Board (decision no: 2025/7095) ethically approved this study.

### Statistical analysis

In this study, demographic, clinical, and laboratory data were compared between patients diagnosed with sepsis and those in the control group. Statistical analysis of the data was performed using IBM SPSS Statistics for Windows, Version 28.0 (IBM Corp., Armonk, NY, USA). Descriptive statistics were summarized using frequencies and percentages for categorical features and median and interquartile range (IQR) for continuous features. Group comparisons, the chi-square test was used for categorical features, and the Mann–Whitney U test, based on distributional assumptions, was used for continuous features. Two-sided hypotheses were considered in all tests, and statistical significance was set at *p* < 0.05.

### Machine learning algorithms and performance evaluation

This section describes the machine learning methods used to evaluate the predictive power of sepsis. The current study developed an explainable machine learning model to predict the risk of sepsis in emergency department patients early. Statistical differences between the control and sepsis groups were examined, and significant increases were observed in the sepsis group in parameters such as the need for intensive care, the presence of positive blood cultures, meeting the criteria for systemic inflammatory response syndrome (SIRS), age, respiratory rate, heart rate, body temperature, leukocyte level, C-reactive protein (CRP), procalcitonin, neutrophil/lymphocyte ratio, and plasma lactate. These findings reveal that sepsis patients have significant differences in physiological and laboratory parameters compared to the control group. To handle missing values, the Random Forest method was used for imputation. The study identified a significant class imbalance problem in the sepsis distribution among 560 sepsis and 1012 non-sepsis patients. This type of imbalance, which is frequently encountered when working with real-world data, can lead to bias towards the majority class and imbalanced learning in machine learning models due to the large number of examples in the majority class compared to the minority class. This can reduce the performance of the model and cause overfitting or underfitting problems. To address this issue, SMOTE-NC was applied in the training data. This method first created a balanced dataset by randomly multiplying the minority class examples. Thus, the model was developed to ensure both overgeneralization and better learning of the minority class. The reason why the SMOTE-NC approach was preferred in the study is that the method is specifically designed for mixed type data (numerical + categorical); that is, it is processed by combining k-nearest neighbor interpolation for numerical features with majority voting for categorical ones. It has been reported in the literature that SMOTE-NC preserves the minority class decision boundaries better than its numerical predecessors alone when categorical predictors are available [13, 14, 15]. The data was divided into two parts, 80% training and 20% testing. In order to ensure the reliability of the models and to report unbiased results, this process was repeated 100 times and the average performance was reported. To determine the best model for sepsis prediction, five different tree-based models (AdaBoost, Gradient Boosting, CatBoost, LightGBM and EBM) were trained and their performances were evaluated. The effectiveness of the models in sepsis prediction was analyzed by calculating the performance metrics of accuracy, sensitivity, specificity, F1 score, and area under the curve (AUC).

## Results

The statistical differences between the control and sepsis groups in the study are evaluated in Table 1. When Table 1 is evaluated, significantly higher rates were observed in the sepsis group in the variables of need for intensive care (*p* < 0.001), presence of positive blood culture (*p* < 0.001) and meeting systemic inflammatory response syndrome (SIRS) criteria (*p* < 0.001). On the other hand, no statistically significant difference was found between the groups in terms of gender (*p* = 0.634) and 28-day survival (*p* = 0.138). In addition, significant increases were determined in inflammation and infection indicators such as age (*p* < 0.001), respiratory rate (*p* < 0.001), heart rate (*p* < 0.001), body temperature (*p* = 0.026), leukocyte level (*p* < 0.001), CRP (*p* < 0.001), procalcitonin (*p* < 0.001), neutrophil/lymphocyte ratio (*p* < 0.001), plasma lactate (*p* < 0.001) in the sepsis group. Hemoglobin level (*p* < 0.001) was significantly lower in the sepsis group. Oxygen saturation (*p* < 0.001) and systolic blood pressure (*p* < 0.001) values were also significantly lower in the sepsis group. These findings reveal that sepsis patients have significant differences in physiological and laboratory parameters compared to the control group (Table 1).

**Table 1:** Descriptive statistics of clinical features by groups

Feature*		Control	Sepsis	*p* value
Gender	Female	443 (43.8%)	254 (45.4%)	0.634
	Male	569 (56.2%)	306 (54.6%)	
Intensive care unit	No	960 (94.9%)	501 (89.5%)	< 0.001
	Yes	52 (5.1%)	59 (10.5%)	
28 days survival	No	48 (4.7%)	35 (6.3%)	0.138
	Yes	964 (95.3%)	525 (93.8%)	
Positve blood culture	Negative	969 (95.8%)	406 (72.5%)	< 0.001
	Positive	43 (4.2%)	154 (27.5%)	
Systemic inflammatory response syndrome	No	312 (30.8%)	99 (17.7%)	< 0.001
	Yes	700 (69.2%)	461 (82.3%)	
Age (years)		68 (25)	76.5 (18)	< 0.001
Systolic blood pressure (mmhg)		136 (31)	130 (36)	< 0.001
Respiratory rate (breaths/min)		22 (6.915)	25.35 (8)	< 0.001
Oxygen saturation (%)		96 (3)	94 (6)	< 0.001
Heart rate (beats/min)		95 (24.625)	100 (26)	< 0.001
Body temperature (°C)		37.8 (1.4)	38 (1.5)	0.031
Hemoglobin (g/L)		132 (24.812)	128 (24)	0.001
Leukocyte particle concentration (×109 cells/L)		11.3 (6.3)	13.1 (7.65)	< 0.001
C-reactive protein(mg/L)		91.5 (120.25)	126 (148.25)	< 0.001
Procalcitonin (ng/mL)		0.13 (0.498)	0.51 (3.88)	< 0.001
Neutrophil–lymphocyte count ratio		8 (9.45)	13.013 (14.3)	< 0.001
Lactate (mmol/L)		1.6 (0.883)	1.9 (1.253)	< 0.001

*: Categorical variables were presented as frequencies (percentages) and quantitative variables as medians (IQR)

In the analysis, the performances of five different machine learning models (AdaBoost, Gradient Boosting, CatBoost, EBM and LightGBM) on the original data and on the data balanced with the SMOTE-NC method were compared. The resampling method was applied to reduce the performance loss caused by the imbalance between the classes, and the results showed that this strategy significantly improved the performance of the models. In all models, remarkable increases were observed especially in F1-score, sensitivity and AUC values, which shows that the minority class can be classified more successfully. While an increase in F1-score from 54.8% to 70.0% and in sensitivity from 50.6% to 70.1% was observed in the AdaBoost model, sensitivity increased from 48.8% to 72.9% and AUC increased from 73.8% to 80.5% in the Gradient Boosting model. Similarly, an increase in F1-score from 55.7% to 76.8% and sensitivity from 49.7% to 77.9% was recorded in the CatBoost model. Although the LightGBM model experienced a slight decrease in specificity, it achieved significant gains in sensitivity and F1-score. In particular, the EBM model achieved the highest success by reaching 79.1% F1-score, 80.9% sensitivity and 84.8% AUC after resampling. The EBM results are an important finding because they combine both the high performance of the model and its explainability. When all these findings are brought together, it is clearly seen that the resampling strategy improves the model performance. When the comparison between the models is made, the EBM model stands out as the strongest model in terms of overall performance, while the CatBoost and Gradient Boosting models are evaluated as other strong alternatives with similar performances (Table 2).

**Table 2:** Model performance comparison on original and SMOTE-NC resampled data

Model*	Accuracy (Orig/Res)	F1 Score (Orig/Res)	Sensitivity (Orig/Res)	Specificity (Orig/Res)	AUC (Orig/Res)
AdaBoost	0.695 / 0.700	0.548 / 0.700	0.506 / 0.701	0.803 / 0.699	0.724 / 0.771
Gradient Boosting	0.704 / 0.723	0.547 / 0.725	0.488 / 0.729	0.829 / 0.717	0.738 / 0.805
CatBoost	0.711 / 0.764	0.557 / 0.768	0.497 / 0.779	0.834 / 0.749	0.759 / 0.837
EBM	0.691 / 0.787	0.533 / 0.791	0.482 / 0.809	0.811 / 0.765	0.720 / 0.848
LightGBM	0.727 / 0.728	0.572 / 0.728	0.497 / 0.728	0.860 / 0.729	0.772 / 0.802

*: In the table, the first value represents the original data and the second value represents the performance metric value obtained after SMOTE

**Fig. 1:**
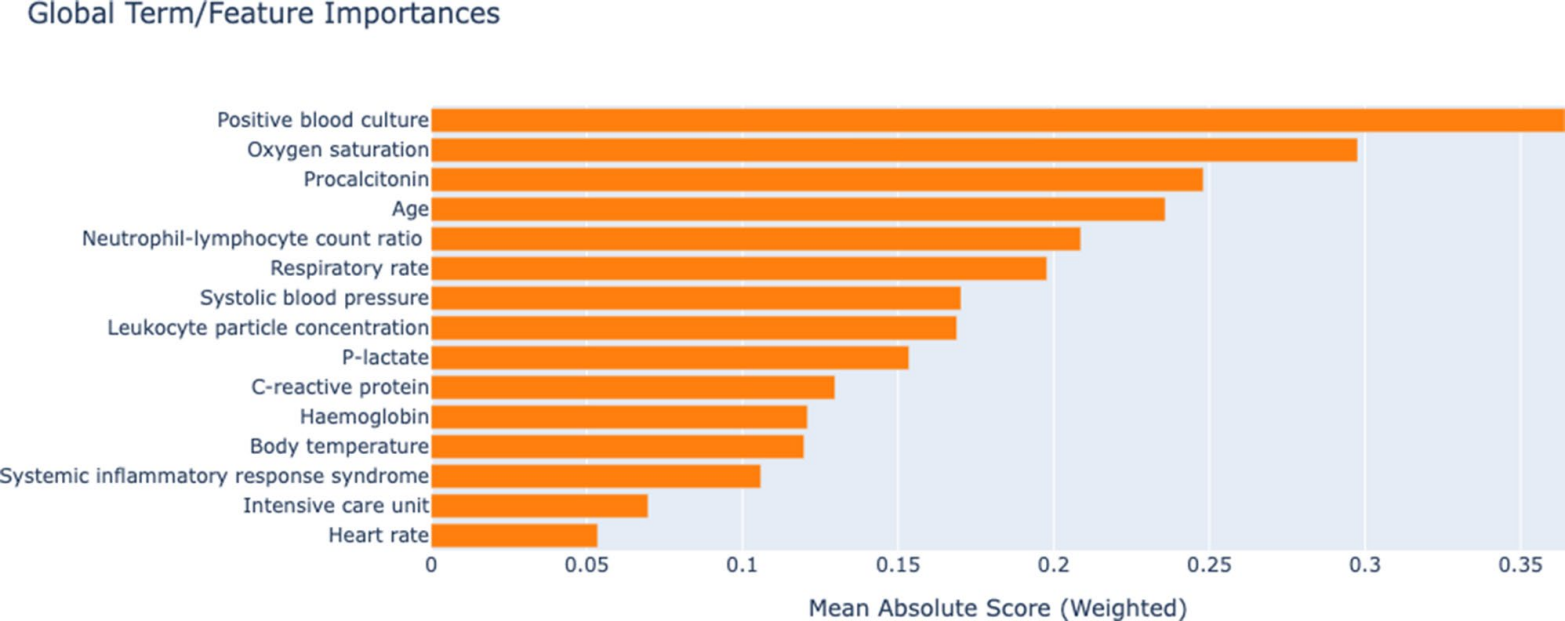
Global interpretation to explain model output

In Fig. 1, the global explanation analysis of the optimal EBM model constructed for sepsis prediction clearly revealed the prognostic contributions of clinical and laboratory parameters. In the global explanations, the variables with the highest weight in the decision process of the model were determined as positive blood culture, oxygen saturation and procalcitonin, respectively. These findings report the critical role of direct evidence of the presence of infection, respiratory function status and procalcitonin on the risk of sepsis. In addition, inflammatory markers such as, age, neutrophil-lymphocyte count ratio and C-reactive protein, as well as vital signs such as systolic blood pressure, respiratory rate and body temperature, were significantly effective in the model. Laboratory parameters such as hemoglobin and leukocyte particle concentration and clinical environment factors (intensive care unit and 28-day survival) also made significant contributions to the model. The transparent structure of EBM quantitatively supports the relationship of these biomarkers with sepsis pathophysiology and clinical management, while indicating that the model can be used as a reliable tool in clinical decision support systems. The results obtained highlight the importance of integrating both dynamic hemodynamic parameters and clinical biomarkers in the multidimensional assessment of sepsis risk (Fig. 1).

**Fig. 2:**
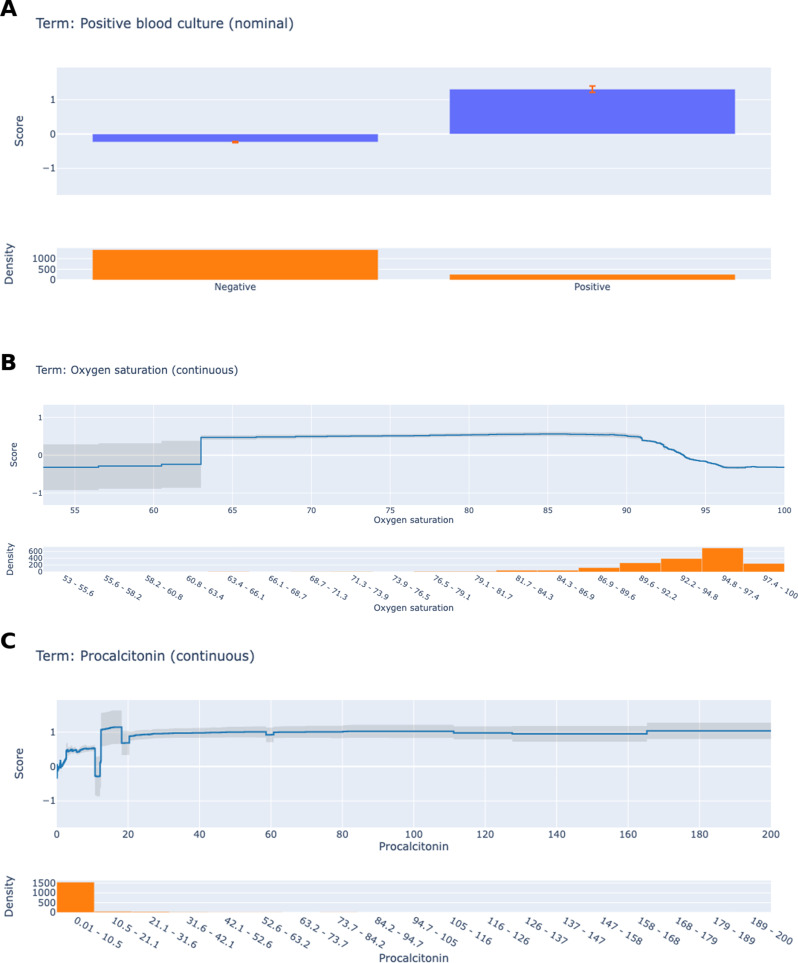
Roles of the top three most important biomarkers in sepsis prediction according to global EBM explanations (Panels **A**, **B**, and **C** illustrate the EBM explanations for positive blood culture, oxygen saturation, and procalcitonin results, respectively)

EBM’s feature shape functions make it possible to display the quantitative contribution of each feature to a single sample. Because each feature’s contribution to an individual’s final forecast can be measured, EBM retains a high level of understandability. Bagging and gradient boosting are used to obtain the complex nonlinear functions that make up each form function. The sequence of the features is irrelevant because the boosting process is limited to training circularly on a single feature at a time with a very low learning rate. The model learns the optimal feature shape function for each feature by iteratively going over them in order to lessen the impact of collinearity. It demonstrates how each feature influences the model’s forecast. Ultimately, the sum of all feature shape functions on the provided characteristics yields the individual final prediction. Figure 2 shows the contribution values to the prediction findings of the top three clinical biomarkers in the global EBM legends. The top line plot for each subfigure displays the features’ contribution, with the estimated accuracy shown by the gray band. The data density of the relevant variable is shown in the bar plot below. The impact of each variable on sepsis risk can be examined by examining the trends of the line graphs of the various variables. In Fig. 2A, the change in the probability of sepsis according to whether the positive blood culture was positive or negative was examined. The results showed that having a positive blood culture increased the probability of sepsis. The oxygen saturation level of the patients was between 53 and 100 and the values were concentrated above 79. When Fig. 2B was examined, it was observed that the probability of sepsis increased when the saturation value was between 79.1 and 89.6 and decreased when it exceeded 89.6 according to the regions where the observations were concentrated. In Fig. 2C, it was seen that the procalcitonin values were concentrated between 0.01 and 31.6. It was determined that the probability of sepsis was lower between the values of 0.01–10.5 and the probability of sepsis increased when procalcitonin reached the levels of 10.5–21.1.

**Fig. 3:**
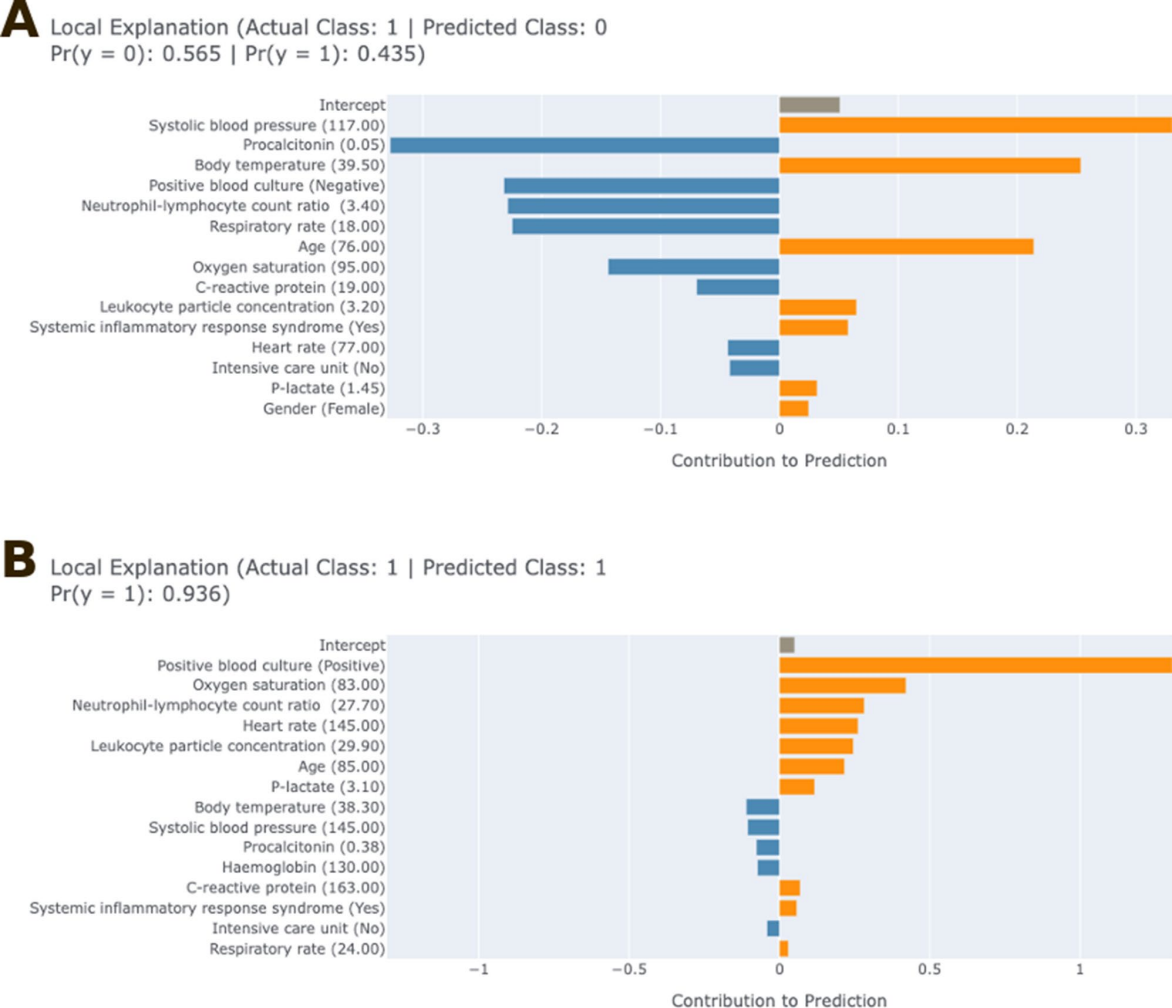
Local EBM explanations for one false negative and one true positive case classified by the model (orange bars represent features contributing positively to the model’s prediction, while blue bars indicate negative contributions. Panels **A** and **B** show the EBM explanation plots for patients with false negative and true positive outcomes, respectively)

The EBM algorithm allows to give detailed contributions of variables for a single prediction. As an example, Figs. 3A and B show the results of a typical false negative and true positive prediction, respectively. In Fig. 3A, the model classified the sepsis patient as a healthy control with 56.5% probability. In terms of the contribution of each clinical biomarker to the predicted model result, variables such as systolic blood pressure, body temperature and age have an effect supporting the predicted result, while procalcitonin, positive blood culture result, neutrophil-lymphocyte ratio and respiratory rate do not support the predicted result. In addition, one of the most dominant effects of the model decision in Fig. 3A is the strong contribution of the normal range systolic blood pressure (117 mmHg) and the very low procalcitonin level (0.05 ng/mL) in favor of the negative class. Remarkably, although the patient had high body temperature (39.5 °C), advanced age (76), and increased neutrophil/lymphocyte ratio, which could clinically suggest sepsis, the model evaluated these contributions to a limited extent and excluded the diagnosis of sepsis. In the Fig. 3B, the model correctly diagnosed sepsis, and in this decision, the strong contribution of classical sepsis findings such as positive blood culture, severe hypoxemia (SpO₂: 83%), markedly high neutrophil/lymphocyte ratio, tachycardia (145 bpm), high β-lactate (3.1 mmol/L), old age, and high C-reactive protein to the positive class is evident. In this patient example, the model correctly recognized the clinical sepsis picture and made a reliable classification. Overall, the EBM model clearly presents which variables are effective in both correct and incorrect classifications, allowing clinicians to interpret model decisions, which is very valuable in terms of model reliability and transparency.

## Discussion

Sepsis is one of the leading causes of mortality and morbidity in patients admitted to the emergency department. This study presents an integrated assessment of clinical and laboratory parameters using XAI models for early prediction of sepsis risk. The findings are particularly notable for the high performance and transparency of the EBM model. In this study, an EBM model was developed for early prediction of sepsis risk in emergency department patients and the performances of different classification algorithms were compared. The results show that the class imbalance problem seriously affects the model performance, but it is possible to overcome this problem with the SMOTE-NC method. In particular, it was observed that the EBM model stands out with higher F1-score, sensitivity and AUC values compared to other models. These findings support the suitability of the EBM model for use in clinical decision support systems due to its high predictive power combined with its explainable structure [16]. In this study, it was found that the need for intensive care, the presence of positive blood culture, and the rates of meeting SIRS criteria were significantly higher in the sepsis group; however, no difference was observed between the groups in terms of gender and 28-day survival rates. The need for intensive care and positive blood culture are critical in the diagnosis of sepsis and in directing treatment protocols [17]. According to the sepsis-3 definition, the need for intensive care is a predictable sign of poor prognosis among patients evaluated through the SOFA score as an indicator of organ dysfunction [2]. The high rate of positive SIRS criteria in our study indicates that SIRS is a sensitive but limited specificity tool in the process where the early inflammatory response turns into sepsis [18]. The high weight of positive blood culture in the model directly indicated that the presence of infection increases the risk of sepsis. The high positive culture rate in this study is an expected result related to severe bacteremia and the presence of invasive pathogens. The fact that the difference between the groups in 28-day survival rates was not found to be statistically significant (*p* = 0.138) should be interpreted due to the sample size and heterogeneous patient characteristics. In our study, the application of early diagnosis and treatment algorithms may have limited the 28-day mortality difference. Significantly higher age, respiratory rate, heart rate, body temperature, leukocyte count, CRP, procalcitonin, neutrophil/lymphocyte ratio and plasma lactate levels in the sepsis group are indicators of systemic inflammation and tissue hypoperfusion. Procalcitonin and lactate in particular are prominent biomarkers in determining both diagnosis and prognosis in sepsis [19]. It has been reported that the diagnostic accuracy of PCT markers is higher than CRP markers in patients hospitalized with suspected bacterial infection [4]. Increased lactate levels are considered a direct indicator of tissue hypoperfusion and are used to monitor treatment response [20]. An increase in the neutrophil/lymphocyte ratio (NLR) is a reflection of the imbalance in the patient’s immune response. In one study, NLR was shown to be an independent predictor of mortality in critical care patients [21]. The findings of this study also support the relevant literature data, and NLR was also included with a high weight in the proposed model. On the other hand, decreases in hemoglobin, oxygen saturation, and systolic blood pressure reveal the negative effects of sepsis on the cardiovascular and respiratory systems [2].

### Model performance and explainability

EBM is an XAI method that offers both high accuracy and understandability. EBM combines security and transparency with shape functions that graphically reveal the contribution of each feature to the decision process [5]. A recent scoping review systematically demonstrated the contribution of feature engineering strategies to performance metrics such as AUROC, sensitivity, and specificity in sepsis prediction models [7]. In addition, Liu et al. [8] demonstrated that dataset diversity strengthens model generalizability by achieving AUC of 0.807 and 0.762 in internal and external validation with the explainable XGBoost model they developed in intensive care patients with traumatic brain injury. Similarly, the interpretable model developed by He and Qiu [9] on the MIMIC-III database provided high calibration and fairness criteria in 28-day mortality prediction with SHAP values. These studies highlight that not only algorithm selection but also integrating data engineering, explainability methods, and model fairness are critical to the success of sepsis early warning systems. In the present study, five different machine learning models (AdaBoost, Gradient Boosting, CatBoost, EBM, and LightGBM) were compared on the original data and the balanced data using the SMOTE-NC method. With the balanced data, the performance of the models showed significant increases, especially in the F1-score, sensitivity, and AUC values. The EBM model exhibited the best performance with the evaluated metrics. These results indicate that machine learning models, especially explainable models, can be effective in predicting sepsis. The global explanations of the EBM model showed that positive blood culture, oxygen saturation, and procalcitonin were the most significant predictors. Feature shape functions visualized the contribution of each feature to the risk of sepsis. For example, the presence of a positive blood culture was shown to increase the probability of sepsis, which is a clinically expected finding. It was observed that the probability of sepsis increased when oxygen saturation was between 79.1 and 89.6 and decreased when it was above 89.6. This is an important finding showing that hypoxia increases the risk of sepsis. It was determined that the probability of sepsis was low when procalcitonin levels were between 0.01 and 10.5 ng/ml and increased when they were between 10.5 and 21.1 ng/ml. This confirms that procalcitonin is a critical biomarker in the diagnosis of sepsis. Among the machine learning models tested in this study, EBM exhibited the highest performance with the evaluated metrics after resampling. These results reflect both the ability of EBM to model complex relationships and its robustness to class imbalance. The dataset balanced with SMOTE-NC provided significant improvements in sensitivity and F1 score, especially for the minority class (patients with sepsis). Similarly, a medical study followed a similar strategy by combining EBM and metabolomics data in acute myocardial infarction and achieved high diagnostic accuracy. This study is important in terms of proving the effectiveness of EBM in a heterogeneous and dynamic clinical picture such as sepsis [16]. The explainable structure of EBM allowed model decisions to be interpreted by clinicians. For example, the determination of positive blood culture, oxygen saturation, and procalcitonin levels as the most critical variables in the prediction of sepsis is consistent with the pathophysiological importance of these parameters. In addition, the contribution of vital signs such as systolic blood pressure and respiratory rate supports the role of hemodynamic instability and organ dysfunction in the diagnosis of sepsis [2]. A different study emphasized that explainable models instead of black-box models increase reliability in clinical decision support systems. In this context, the transparency of EBM strengthens its application potential by ensuring that clinicians trust the model outputs [22]. The contribution of each feature to individual prediction is numerically revealed through the shape functions of EBM; thus, clinicians can understand “why” and “how” the model makes decisions. This has the potential to increase the adoption rate of clinical decision support systems by eliminating the “confidence crisis” common in black-box models [6]. Although the results are promising, it is important to interpret them with caution due to the absence of external validation. Nonetheless, the repeated 100-fold validation strategy and the explainable nature of the EBM model strengthen the internal validity and clinical interpretability of the current findings. To address the critical need for external validation, we will prioritize prospective multicenter trials across diverse patient populations and healthcare systems. Collaborations with international clinicians will ensure the model’s adaptability to varying clinical workflows, EHR infrastructures, and demographic profiles [10]. Prospective studies will follow standardized frameworks, such as the SPIRIT-EHR guidelines, to rigorously assess the model’s real-world efficacy, including metrics like reduced false negatives, improved early intervention rates, and clinician satisfaction. Furthermore, iterative co-design sessions with frontline physicians will refine model outputs to align with triage priorities, optimize alert thresholds, and minimize alarm fatigue. These efforts will bridge the gap between algorithmic predictions and clinical trust, ensuring the model’s integration into high-stakes decision-making processes [11].

### Class imbalance and sample size

The fact that the number of patients with sepsis (*n* = 560) was lower than those without sepsis (*n* = 1012) in the study led to class imbalance, which is a common problem in machine learning models. Overcoming this problem with SMOTE-NC provided up to 20% increase in sensitivity, especially in AdaBoost and Gradient Boosting models. Similarly, a paper reported that SMOTE-based methods improved the classification of the minority class in diabetes diagnosis. However, SMOTE-NC’s generation of synthetic examples may be insufficient to represent rare extreme values in real-world data. This may limit the generalization performance of the model. In this study, this risk was partially offset by using cross-validation and an external test set [23]. The sample size (*n* = 1572) can be considered as a strength in terms of increasing statistical power. In this study, class imbalance was significantly reduced by using the SMOTE-NC method; In this way, significant increases were achieved in the criteria such as sensitivity, F1-score and AUC of the models. For example, the F1-score of the AdaBoost model increased from 54.8% to 70.0%; the AUC of Gradient Boosting increased from 73.8% to 80.5%; and the CatBoost and EBM models exhibited similar gains. These results show that eliminating the imbalance in medical data with imbalanced classes provides significant benefits to the models, similar to the increases obtained with methods such as SMOTE or ADASYN in the literature [24, 25]. Thus, reducing the false negative rate in the early diagnosis of a critical disease such as sepsis is of vital importance for patient safety. SMOTE-NC’s effectiveness in improving minority-class learning (e.g., sepsis cases) is notable, achieving a 30.7% F1-score increase over unbalanced data. However, synthetic oversampling carries risks of unrepresentative samples, as highlighted in prior studies [24, 25]. By preserving biological relevance in key biomarkers (e.g., procalcitonin, blood culture), our approach mitigates this limitation compared to generic resampling methods. SMOTE-NC is particularly suited for this type of medical data because it handles categorical and continuous features separately. It uses k-nearest neighbor interpolation for numerical values (like procalcitonin) and majority voting for categorical features (like positive blood culture status). This dual approach helps ensure that the synthetic samples represent plausible clinical scenarios rather than mathematically possible but biologically irrelevant combinations of features.

### The role of clinical and biomarkers

Significant increases in procalcitonin, CRP, and lactate levels in the sepsis group confirm the central role of these biomarkers in the infection and inflammation response [2]. On the other hand, low hemoglobin and oxygen saturation indicate that tissue hypoxia and anemia accompany patients with sepsis. These findings are similar to inflammatory processes associated with metabolic reprogramming in rheumatoid arthritis [26]. Local explanations of EBM revealed that low procalcitonin and normal systolic blood pressure affected the model decision in a false-negative case. This situation emphasizes that clinical findings may contradict each other and that models require multidisciplinary evaluation. Local explanations of EBM showed that the model sometimes made a decision despite the contradictions between clinical findings. In a false-negative case, normal systolic blood pressure and low procalcitonin values made a strong contribution in favor of the model decision; However, it was observed that classifier findings such as high body temperature and increased neutrophil/lymphocyte ratio in the patient were weighted relatively weaker by the model. This situation emphasizes that machine learning models alone cannot completely replace clinical decision making; a multidisciplinary approach and physician supervision are still indispensable [5]. These inconsistencies in clinical data can be minimized with harmonized protocols and human-machine collaboration. In addition, cytokines such as IL-6 and TNF-α as well as markers of endothelial dysfunction have important prognostic value in the immunopathological process of sepsis. It has been shown that angiopoietin-2 levels make a significant contribution, independent of CRP and procalcitonin, in predicting the risk of organ failure and mortality in septic patients [27]. In addition, it has been proven that keeping the lactate clearance rate, which evaluates tissue hypoperfusion, above 10% in the first 6 h significantly reduces the mortality rate in patients with septic shock. These findings suggest that sepsis is a multidimensional disease; rather than relying on just one or two biomarkers, combined analysis of cytokine profile, endothelial function parameters, and metabolic response indicators will provide a more holistic approach to the real-time status of the patient. Thus, these additional biomarkers that can be added to machine learning models can further increase the sensitivity and specificity performance of the model [3]. These advanced inflammatory markers (e.g., IL-6, TNF-α, angiopoietin-2) were not included in the present analysis due to their absence in the publicly available, retrospective dataset utilized for this study. As these tests are not part of routine emergency department workups in many institutions, their inclusion was not feasible.

### Clinical applications and future studies

This study demonstrates the potential of EBM as a tool to support early diagnosis in patients with suspected sepsis in the emergency department. Real-time integration of the model can optimize triage processes and increase the chance of timely intervention. However, the model needs to be tested in different patient populations (e.g., pediatric, immunocompromised) and with multicenter data. In addition, continuous updating of the model with dynamic data flow will be critical to maintain performance. In future studies, the addition of proteomic and genomic data to the model may contribute to the determination of sepsis subtypes. Similarly, a medical study has shown that the integration of multi-omics data in breast cancer metastasis increases prognostic value [28]. In addition, prospective clinical studies should be planned to measure the impact of model decisions on patient outcomes. This study has demonstrated the potential to optimize sepsis diagnosis and classification processes with machine learning-supported systems. Explainable models, especially EBM, can be used as decision support tools in clinical settings; they offer the opportunity to improve patient care when combined with early warning (alert) systems. In the future, real-time electronic health record system (EHR) integrations and live clinical tests of prototypes will reveal the model’s ability to learn and adapt over time. In addition, the inclusion of “omics” data such as genomics and metabolomics will open new doors for identifying molecular subtypes of sepsis and personalizing treatment strategies. Ultimately, the synthesis of clinical expertise with machine learning will provide significant progress towards improving patient outcomes in the management of complex diseases such as sepsis. Future work should focus on validating the model on external and multicenter datasets to assess generalizability. Collaborations with clinicians from different institutions will be essential to ensure the model’s relevance across diverse settings. Additionally, prospective clinical studies can provide evidence on real-time applicability and its effect on clinical outcomes, such as reduced time-to-intervention or mortality. Developing pipelines for real-time EHR integration and monitoring alert fatigue should also be prioritized. Also, real-time integration of the model into electronic health record systems could optimize triage workflows by automating risk scores and triggering clinician alerts. Additionally, incorporating multi-omics data (e.g., genomics, metabolomics) may enable subtype-specific sepsis prediction, aligning with precision medicine goals. Multicenter validation across diverse populations will further assess generalizability and adaptability to varying clinical environments. For practical implementation, the model would require real-time access to a specific set of EHR data points, including vital signs, standard laboratory results (e.g., CBC, CRP, procalcitonin), and blood culture status. The output could be presented to clinicians as an integrated ‘Sepsis Risk Score’ within the patient’s main EHR dashboard. To maximize utility and trust, this score could be clickable, leading to the EBM’s local explanation plot, which visually details the specific factors contributing to the patient’s current risk assessment. A concrete multi-stage validation plan would include: (1) retrospective validation on datasets from different institutions with diverse patient demographics, (2) a prospective observational study to assess real-time performance without influencing clinical care, and (3) a prospective interventional trial to measure the model’s impact on clinical outcomes, guided by established frameworks like SPIRIT-AI and CONSORT-AI.

### Study limitations

The retrospective design of the study may introduce potential bias during the data collection process. Additionally, the single-center dataset may limit the validity of the model in other geographies or different healthcare systems. Due to the retrospective and single-center nature of the dataset, the generalizability of the model to different hospital settings, EHR systems, or clinical workflows may be limited. This limitation is particularly important given that model performance can vary significantly across institutions with different patient demographics and clinical practices. SMOTE-NC’s generation of synthetic samples carries the risk of being unrepresentative of rare clinical scenarios. Finally, the model’s performance is dependent on laboratory parameters, which may make it difficult to apply in resource-limited settings.

## Conclusion

This study offers three principal contributions to the existing literature, effectively addressing a notable research gap. First, it introduces an interpretable, EBM-based machine learning framework specifically designed for the early prediction of sepsis in emergency department settings. Second, it tackles the critical issue of class imbalance between sepsis and control groups by employing the SMOTE-NC method, resulting in a substantial improvement in sensitivity and F1-score metrics compared to the original imbalanced data. Third, it enhances clinical utility by providing a dual-level interpretability approach that elucidates model decisions both globally (overall model behavior) and locally (individual predictions), thereby offering actionable insights to clinicians. In contrast to many existing studies that rely on black-box algorithms or are derived from intensive care unit data, our research distinguishes itself through its emphasis on model transparency, direct clinical applicability, and rigorous validation via robust replication methods. Unlike ‘black-box’ models or those trained on ICU data, our ED-focused framework is designed for the unique constraints and needs of emergency care. By offering a reliable and understandable decision-support tool, this research represents a significant step towards integrating trustworthy AI into frontline clinical practice to improve sepsis outcomes.

## Data Availability

The raw data supporting the conclusions of this article will be made available by the authors on request.
